# Acknowledgement to Reviewers of *Nanomaterials* in 2018

**DOI:** 10.3390/nano9010108

**Published:** 2019-01-17

**Authors:** Nanomaterials Editorial Office

**Affiliations:** MDPI, St. Alban-Anlage 66, 4052 Basel, Switzerland

Rigorous peer-review is the corner-stone of high-quality academic publishing. The editorial team greatly appreciates the reviewers who contributed their knowledge and expertise to the journal’s editorial process over the past 12 months. In 2018, a total of 1092 papers were published in the journal, with a median time to first decision of 13 days and a median time to publication of 30 days. Among the reviewers who reviewed for *Nanomaterials* in 2018, the following five have been selected to receive the “*Nanomaterials* 2018 Outstanding Reviewer Awards” for the timeliness and quality of their review reports in 2018:

Lazzara, Giuseppe from University of Palermo, Italy
Ariga, Katsuhiko from National Institute for Materials Science Tsukuba, Japan
Szekely, Gyorgy from University of Manchester, UK
Cavallaro, Giuseppe from University of Palermo, Italy
Rodriguez, Noel from University of Granada, Spain

The editors also would like to express their sincere gratitude to the following reviewers for their cooperation and dedication in 2018:

Abad, Manuel DavidLee, JaejongAbashev, George G.Lee, Jae-SukAbdul Samad, YarjanLee, JungwooAbou Neel, Ensanya A.Lee, SejoonAbramovich, HaimLee, Seung HeeAbyaneh, Majid KazemianLee, SunwooAchelle, SylvainLee, WooyoungAdamcakova-Dodd, AndreaLee, Young-ChulAdamcik, JozefLeem, Jung WooAdlhart, ChristianLefebvre, David E.Afanasev, AndreiLéger, BastienAgarwala, ShwetaLegrand, François XavierAgatemor, ChristianLeitao, Diana C.Agnoli, StefanoLeitner, MichaelAgonafer, DerejeLekatou, A.Agrawal, RichaLeliaert, JonathanAhmad, ZeeshanLellouche, Jean-PaulAhmadivand, ArashLembrikov, BorisAhmed, KhalilLeonidas, PalilisAhmed, MaryaLeporatti, StefanoAkens, MargareteLepore, MariaÅkermark, BjörnLerouge, FredericAlastalo, AriLesch, AndreasAl-Bataineh, SameerLesiak, PiotrAlbu, Ana MariaLesz, SabinaAlcoutlabi, MatazLétoublon, AntoineAldakov, DmitryLevacher, DanielAlegre, CinthiaLevchenko, IgorAlegria, Elisabete C. B. A.Levine, MindyAlessandri, IvanoLewandowski, WiktorAlexandrou, Antigoni Lezama, LuisAlexiou, ChristophLhuillier, EmmanuelAlexis, FrankLi, JiashenAlhabeb, MohamedLi, LongyuAlhalawani, AdelLi, Lu HuaAljabali, AlaaLi, MingqiangAllongue, PhilippeLi, WeiyangAlMangour, BandarLi, YingAlmeida, Bernardo G.Liakos, IoannisAlmeida, Maria Adelaide AraújoLiang, Chi-TeAlmoazen, HassenLiang, LiuenAlongi, JennyLiang, YucangAltomare, MarcoLiao, Jiunn-DerAlvarado, VladimirLiberti, MicaelaAlvarez, J. IgancioLichtenegger, HelgaÁlvarez, MercedesLi-Destri, GiovanniAlvarez-Alonso, PabloLiguori, AnnaAlvarez-Dominguez, CarmenLikodimos, VlassisAlzobaidi, ShehabLillo-Rodenas, Maria AngelesAmabili, MarcoLim, Dong ChanAmarandei, GeorgeLim, Eun-KyungAmaro, Ana Martins Lin, Hung-YinAn, KwangjinLin, LouisAn, SeongpilLin, Yang-weiAnanikov, Valentine P.Lin, YuqingAnanthakrishnan, Soundaram JeevarathinamLin, YuyuanAnasori, BabakLinert, WolfgangAndersen, Christian P.Ling, Yong-ChienAnderson, ScottLinko, VeikkoAndò, BrunoLinul, EmanoilAndou, YoshitoLionetto, FrancescaAndreani, TatianaLiopo, AntonAndrés, JuanLiskiewicz, TomaszÁngel Sogorb , Miguel List-Kratochvil, Emil J.W.Angelova, AngelinaLiu, BinAnggraini, Sri AyuLiu, LihongAngilella, Giuseppe G. N.Liu, QingkunAnni, MarcoLiu, RenAnvari, BahmanLiu, Shyh-JiunAo, ZhiminLiu, XiangshengAoni, Rifat AhmmedLiu, YanbiaoAoyagi, TakaoLiu, ZhiqiuApetrei, ConstantinLizundia, ErlantzAppelhans, DietmarLlobet, EduardAppiah-Hagan, EmmnauelLloveras, PolAprea, CiroLo, Kuang YaoAradilla, DavidLoja, Maria A. R.Arakawa, TakahiroLojou, ElisabethAraña-Mesa, JavierLonghi, MariangelaAranda, Miguel A. G.Lopes, Maria AscensãoArandiyan, HamidrezaLopez Acevedo, OlgaAraujo, DanielLópez De Lacalle, Luis N.Ardizzone, SilviaLopez, ReneArena, FrancescoLópez-Garzón, F. J.Arges, ChristopherLópez-Lorente, Ángela InmaculadaAriga, KatsuhikoLópez-Ortega, AlbertoAriño, CristinaLorenzi, AndreaArmbruster, UdoLorenzo, EncarnaciónArmentano, IlariaLosurdo, MariaArnusch, Christopher J.Lotnyk, AndriyArosio, PaoloLoureiro, Jose MiguelArpicco, SilviaLove, Corey TArrieta, MarinaLu, Ming-ChangArrigo, RossellaLu, YaoArroyo, NetzahualcóyotlLub, JohanArunachalam, PrabhakarnLubal, PřemyslArvanitis, KonstantinosLuber, SandraArvapally, Ravi KumarLucena, RafaelAsadnia, MohsenLuciani, GiuseppinaAsefa, TewodrosLukac, PavelAskari, HassanLukyanov, PavelAta, SeisukeLuong, John H. T.Atabaev, TimurLutz, MartinAtchudan, RajiLützenkirchen, JohannesAtuchin, Victor V.Lützenkirchen-Hecht, DirkAvgouropoulos, GeorgeLv, KangleAw, KeanLyalin, AndreyAyers, John E.Lyons, JohnAzarang, MajidLyutakov, OleksiyBabicheva, ViktoriiaMa, LuBabu, RameshMa, Sheng-xingBachmann, MartinMa, TianyiBaek, Jong-SuepMaas, MichaelBaeza-Squiban, ArmelleMacak, Jan M.Balalaeva, IrinaMacdonald, ThomasBalandin, Alexander A.Machrafi, HatimBalasingam, Suresh KannanMachut-Binkowski, CécileBaldi, FrancoMadureira, Ana RaquelBaldoví, Jose J.Maestre, JorgeBallirano, PaoloMaggioni, DanielaBalme, SébastienMahata, Manoj KumarBalzer, FrankMahony, Timothy JohnBandala, Erick R.Maia, FredericoBandiello, EnricoMaisch, TimBanfi, FrancescoMajak, JüriBarba, Francisco J.Majewski, LeszekBarba, MartaMakarona, E.Barbetta, AndreaMakihara, KanjuroBarbier, A.Makise, KazumasaBarbillon, GrégoryMalhotra, RajivBardi, GiuseppeMalinauskas, MangirdasBarelle, CarolineMalka, DrorBarille, RegisMalkin, AlexanderBarnes, Frank S.Mallavia, RicardoBarretta, RaffaeleMallidi, SrivalleeshaBarron, AndrewMalucelli, GiulioBar-Sadan, MayaMamistov, A. I.Bartolomeo, Antonio DiManca, Maria LetiziaBasit, Munshi MahbubulManera, Maria GraziaBaskoutas, SotiriosMani, Ganesh KumarBastarrachea, Luis J.Manian, Avinash P.Bastola, EbinMannino, SaverioBatmunkh, MunkhbayarMansoori, G. AliBatra, A. K.Mao, ChuanbinBattaglia, EricMao, XiaoanBauer, WolfgangMarasso, Simone LuigiBayer, IlkerMarcelli, Augusto ClaudioBazylińska, UrszulaMarchesan, SilviaBeall, GaryMarchini, CristinaBeard, James D.Marconcini, PaoloBeata, Kalska-SzostkoMardare, Andrei I.Beattie, James K.Marino, AttilioBedia, JorgeMarken, FrankBégin, DominiqueMarrani, AndreaBehzadi, ShahedMarrocchi, AssuntaBella, FedericoMarsal, Lluis F.Bellan, Leon M.Martínez, CristinaBellet, DanielMartínez-Espinosa, Rosa MaríaBeneventi, DavideMartinez-Triguero, JoaquinBenito, José ManuelMartino, MarcoBennici, SimonaMartino, SabataBensaoula, AbdelhakMartín-Ramos, PabloBernal, M. MarMartins, Luísa MargaridaBernardi, Rafael C.Martins, RodrigoBernardo, LuísMartins, Rui C.Berret, Jean-FrançoisMastrangeli, MassimoBert, NikolayMastropasqua, LeonardoBertoncello, PaoloMatějka, VlastimilBesara, TigletMaterer, Nicholas F.Bessergenev, Valentin G.Mathieu, DidierBettazzi, FrancescaMatsui, DaisukeBhattacharjya, DhrubajyotiMauriello, FrancescoBhattacharya, SriparnaMauriz, ElbaBianchi, MassimilianoMavrogonatou, EleniBianco, I. D.Mazzaglia, AntoninoBiernat, JanMazzer, MassimoBilenberg, BrianMcCord, JeffreyBilewicz, AleksanderMcGilly, LeoBiondi, MarcoMcLean, AlexanderBirch, BrianMcMahon, Jeffrey M.Birch, DavidMcMillan, PaulBisio, FrancescoMei, BastianBittkau, KarstenMeier-Menches, Samuel M.Bjorklund, Robert B.Meijide, FranciscoBjörkman, TorbjörnMeilhan, JéromeBlanchard, PaulMejia, IsraelBlanco, AngelesMele, ElisaBlanco, IgnazioMelin, FredericBlayo, AnneMélinon, PatriceBloh, Jonathan Z.Memoli, GianlucaBludov, YuliyMénard-Moyon, CéciliaBochenkov, VladimirMéndez, BianchiBoisselier, ÉlodieMeng, LiJianBojková, BiankaMeng, XiangboBoldt, RegineMeng, XiangchaoBombelli, PaoloMériguet, GuillaumeBon, VolodymyrMerlino, AntonelloBorges, João PauloMestre, Ana SofíaBormashenko, EdwardMeulenberg, ElineBoschker, JosMeulenberg, Robert W.Bosi, SusannaMeziani, MohammedBossmann, StefanMezzi, AlessioBouazizi, NabilMiah, Abdul HalimBoucher, David S.Michalska-Domańska, MartaBoudaden, JamilaMichel, Carlos R.Bourillot, EricMickelson, AlanBousbaa, HassanMiculescu, FlorinBoutinguiza, MohamedMiecznikowski, KrzysztofBowen, ChrisMilanesio, MarcoBrameshuber, MarioMilčius, DariusBranda, FrancescoMildren, RichBrankovic, StankoMills, DavidBrantley, William A.Milone, CandidaBreynaert, EricMilosevic, IrenaBrezesinski, TorstenMilot, Rebecca L.Briand, DanickMin, KimBriedis, VitalisMinakshi, ManickamBrieger, JuergenMinea, Alina AdrianaBrooks, Donald E.Mingareev, IlyaBrosseau, ChristianMiola, MartaBrown, DavidMiranda, Jose M.Brühwiler, DominikMiranda-Castro, RebecaBrundo, Maria ViolettaMiras, Haralampos N.Brunner, AndreasMiroshnichenko, AndreyBrutti, SergioMiseki, YugoBuchelnikov, VasiliyMishra, Pawan KumarBufo, Sabino AurelioMishra, Yogendra KumarBugla-Płoskońska, GabrielaMitchell, JohnBuha, JokaMitchell, Scott G.Bünzli, Jean-ClaudeMitrakas, Manassis M.Buonocore, FrancescoMiyazaki, ToshikiBurlacu, AlexandrinaMizsei, JánosBurns, Jonathan D.Mizuno, MasashiBusquets, Maria AntòniaMkandawire, MartinBussy, CyrillMobed-Miremadi, MaryamButté, RaphaëlMohanta, AntaryamiBystrov, VladimirMohd-Yasin, FaisalCabaj, JoannaMohles, VolkerCabaleiro, DavidMohn, DirkCaballero, AlvaroMokaya, RobertCabot, AndreuMomeni, KasraCabrales, LuisMonclus, MichelCabulis, UgisMondal, KunalCacciotti, IlariaMonflier, EricCadatal-Raduban, MarilouMontenegro, LuciaCahay, Marc M.Montes Bayón, MaríaCalabrese, LuigiMonzón, AntonioCaldas, PauloMorales, JuliánCamarillo, RafaelMorales-Garcia, AngelCamblor, Miguel A.Moratti, SteveCamerel, FranckMorávková, ZuzanaCameron, DavidMorbidelli, MassimoCameron, JosephMoreno, MiguelCametti, CesareMorganti, PierfrancescoCamilli, LucaMorley, Nicola A.Caminati, GabriellaMouchet, SébastienCampanella, LuigiMourdikoudis, StefanosCampbell, MichaelMousa, ShakerCampioni, SilviaMousseau, F.Caneschi, AndreaMozafari, M. R.Cannio, MariaMozaffari, SaeedCannone Falchetto, AugustoMozetič, MiranCao, YuheMrówczyński, RadosławCapek, LiborMrowiec-Białoń, JulitaCaputi, LorenzoMuench, FalkCaputo, DomenicoMuguruma, HitoshiCaputo, Gregory A.Muhammad-Sukki, FirdausCarabineiro, SóniaMüller, GerhardCaramella, Carla M.Müller, KarstenCarbonnier, BenjaminMüller, TimoCárdenas, SoledadMuñoz-Batista, Mario JesúsCardia, Maria CristinaMunteanu, Florentina-DanielaCarlen, Edwin T.Münzenrieder, Niko S.Carneiro, Olga SousaMusumeci, TeresaCarosio, FedericoNabae, YutaCarreon, MoisesNácher, AmparoCasari, CarloNag, ManojCaseri, Walter RemoNagai, MasayaCassagnau, PhilippeNagao, DaisukeCassidy, John F.Nagatani, NaokiCastaldi, GiuseppeNagraj, VinayCastanheira, Elisabete M. S.Naha, Pratap C.Castanheiro, JoséNakagawa, YoshinaoCasula, GiuliaNakajima, AnriCasula, Maria F.Nakamura, AtsutomoCauchy, ThomasNakamura, KeisukeCavallaro, GiuseppeNakamura, TakashiCavallini, MassimilianoNakashima, NaotoshiCazorla-Amorós, DiegoNanda, HimansuCebers, AndrejsNandwana, VikasCecilia, Juan AntonioNarciso, JavierCelano, UmbertoNattestad, AndrewCelorrio, VeronicaNaumov, AntonCeresa, BrianNavarro, Jorge A. R.Cerrato, GiuseppinaNavarro, María VictoriaCesano, FedericoNavío, J. A.Chai, Kyu YunNayak, ArunimaChakrapani, VidhyaNazare, ShonaliChalker, Justin M.Ndieyira, JosephChang, Chia-ChenNees, MatthiasChang, Chih-YuNegishi, NobuakiChang, LingqianNegro, EnricoChang, Sheng-PoNemes, Norbert M.Chang, Yao-FengNemnes, George AlexandruChantana, JakapanNeveu, SophieCharas, AnaNgo, Hoan ThanhCharilaou, MichalisNguyen, Duc ThanhChase, George G.Nguyen, Nhat TruongChava, Rama KrishnaNiedziela, TomaszChavarin, Carlos Alvarado Niemirowicz-Laskowska, KatarzynaChen, Chih-MingNikas, George K.Chen, Chuh-YungNikiforov, AntonChen, ChunhuiNikolelis, Dimitrios P.Chen, HongweiNilsen, OlaChen, Jem-KunNima, Zeid A.Chen, Jian-LianNishihara, HirotomoChen, JunNisticò, RobertoChen, JunNistor, CristinaChen, LongNiu, Catherine H.Chen, Pang-ShiuNogues, JosepCheng, Fong-YuNohe, AnjaCheng, PeiNorton, MichaelCheng, XingguoNoubactep, ChicgouaCheong, In WooNouranian, SasanCheruzel, LionelNovio, FernandoChianese, AngeloNovotna, VladimiraChiappini, AndreaNtalli, Nikoletta G.Chiappone, AnnalisaNunes, Carla D.Chiavaioli, FrancescoNurunnabi, MdChikan, ViktorNypelö, TiinaChinga-Carrasco, GaryO’Rear, EdgarChiolerio, AlessandroObraztsov, AlexanderChizhik, Alexey I.O'Brien, LiamChmelik, FrantisekOchiai, TsuyoshiCho, Cheong-WeonOćwieja, MagdalenaCho, Dae WonOgino, ToshioCho, In SunOh, Daniel S.Cho, SungboOh, Dongyeop X.Cho, Sung-JinOh, Soong JuChoi, Chul-JinOkamoto, MasamiChoi, HyoungOkochi, MinaChoi, JungwookOlivares, JimenaChoi, Myong YongOliveira, Paula A.Choi, Nag JungOlivera-Pastor, PascualChoi, Sang-IlOlmos, DaniaChou, Shih-FengOlsen, ThomasChow, JamesO'mullane, AnthonyChrissopoulou, KiriakiOnbasli, M. C.Christensen, Jørn B.Opitz, JörgChroneos, AlexanderOrdejón, PabloChruściel, Jerzy J.Órfão, J. J. M.Chrysikopoulos, Constantinos V.Ori, OttorinoChrzanowski, WojciechOrte, AngelChul Wee, LeeOrtega, José MarcosChun, Heung JaeOrtiz, Gregorio FChung, Eun JiOrtolani, MicheleChung, Sheng-HengOschatz, MartinChung, YongseogOta, SatoshiCicala, GianlucaOuerghi, AbdelkarimCiciliot, StefanoPacurari, MaricicaCiesielski, MichaelPadil, Vinod V. T.Ciofani, GianniPagliara, StefanoClement, JoachimPala, NezihClimente, Juan I.Palamà, IlariaCohen, Sidney R.Palasantzas, GeorgeCoisson, MarcoPalchoudhury, SoubantikaColace, LorenzoPalinko, IstvanColangelo, FrancescoPallandre, AntoineColangelo, Gianpieropalma, matteoColavita, PaulaPalmas, SimonettaColeman, Anthony WilliamPalocci, CleofeCollins, ScottPalomo, JoseColmenares, Juan CarlosPalys, BarbaraColombi, PaoloPalzer, StefanComelli, DanielaPan, QinComfort, K. K.Pan, Tung-MingCompañ, VicentePan, ZhuComparelli, R.Panomsuwan, GasiditConcilio, SimonaPantazopoulou, StavroulaConstantino, FerdinandoPanzarini, ElisaConti, BicePaolicelli, GuidoCoppari, FedericaPapadimitriou, VassilikiCorat, Evaldo JoséPapavassiliou, DimitriosCornell, B.Papp, AndrásCorsi, IlariaParajuli, DurgaCortese, BarbaraParak, WolfgangCortesi, RitaPardanaud, CédricCosco, DonatoParedes, José ManuelCosma, PinalysaParigger, ChristianCosta Lima, SofiaParigger, Christian G.Costa, Carlos M.Parisi, FilippoCostantino, LucaPark, Bong JooCoudert, Francois-XavierPark, Byung-WookCouillaud, FranckPark, Chul-hoCoutinho, Paulo José GomesPark, Dong HyukCran, Marlene J.Park, In-KyuCristea, CeciliaPark, JonghyunCritchley, KevinPark, Soo-JinCroce, Anna CletaPark, SunggookCroissant, JonasPark, Yong IlCruz-Silva, RodolfoParmentier, JulienCtistis, GeorgiosPasán, JorgeCucinotta, FabioPasini, DarioCui, BoPaspalakis, EmmanuelCui, HaitaoPasqua, LuigiCuomo, FrancescaPassaglia, ElisaCustódio, CatarinaPasternak, GrzegorzCzech, BożenaPastero, LindaCzekanski, AleksanderPastore, RobertoCzernecki, RobertPastrana-Martínez, Luisa M.Czujko, TomaszPatel, ManuDabkowska, Hanna A.Pathakoti, KavithaDabrowiak, JamesPati, RanjitDacarro, GiacomoPatterson, JenniferD'Accolti, LuciaPatterson, JenniferDaftarian, PirouzPauliac-Vaujour, EmmanuelleDale, Phillip J.Pauliukaite, RasaDale, SaraPavlík, ZbyšekDalmoro, AnnalisaPayam, Amir FarrokhDamjanovski, SashkoPazos-Perez, NicolasD'Andrea, CristianoPeana, Massimiliano F.Däne, MarkusPegoretti, AlessandroDantan, AurélienPeijnenburg, WillieDarafsheh, ArashPeisert, HeikoDarmanin, ThierryPeña, Maria Marjorette O.Das, JagotamoyPeña-Méndez, Eladia M.Dash, BirajaPeng, HongDavid, LamuelPensabene, VirginiaDavis, FrankPeppel, TimDavis, FredPerche, FédéricoDawn, ArnabPereira, EulaliaDe Aza, Piedad N.Pereira, Luiz De Bonis, AngelaPérez, Esther EnríquezDe Coninck, Joel Pérez-Gago, María BernarditaDe Geyter, NathaliePérez-Mendoza, Manuel J.de la Presa, PatriciaPeriana, Roy A.De La Torre, UnaiPerzynski, RégineDe Marco, RossellaPetcu, CristianDe Marcos Ruiz, SusanaPetersen, ElijahDe Rosa, Maria CristinaPetit, TristanDe Stefano, LucaPetkov, NikolayDebliquy, MarcPetukhov, Andrei V.Deimede, ValadoulaPezzella, AlessandroDeitzel, JosephPhan, Hoang-PhuongDel Pino, PabloPhilipose, Ushadel Puerto Morales, MariaPhuoc, Nghia TruongDella Gaspera, EnricoPiana, GiuliaDell'Agli, GianfrancoPicca, Rosaria AnnaDell'Olio, FrancescoPiecek, WiktorDelogu, LuciaPielichowski, KrzysztofDelville, Marie-HélènePilot, RobertoDemel, JanPimentel, AnaDeng, GuochuPinchuk, AnatoliyDenizot, Benoit Pineda, AntonioDennis, James E.Pineda, Teresa Derosa, PedroPineiro, Manuel M.Devel, MichelPiñeiro, MartaDhakal, AshimPinho, LuísDi Bartolomeo, AntonioPini, ValerioDi Bella, ClaudiaPinto, Artur M.Di Bella, SantoPiperno, AnnaDi Credico, BarbaraPires, José Carlos MagalhãesDi Girolamo, NickPirzada, TahiraDi Martino, PieraPispas, StergiosDi Mauro, AlessandroPistone, AlessandroDi Palma, Luca Pizzagalli, LaurentDi Tommaso, DevisPizzoferrato, RobertoDiamanti, Maria VittoriaPlaczek, MarekDiao, ShuoPlakas, KonstantinosDíaz-García, Marta ElenaPłotka-Wasylka, JustynaDickert, Franz L.Pohl, PawelDieringa, HajoPolat, Emre OzanDíez-Pascual, Ana MaríaPolavarapu, LakshminarayanaDilonardo, ElenaPolimeni, AntonellaDimakis, NicholasPolimeno, AntoninoDimitratos, NikolaosPolitano, AntonioDimos, KonstantinosPolyak, BorisDina, Nicoleta ElenaPoncet, SébastienDinesh, BhimareddyPons, JosefinaDinischiotu, AncaPonzoni, AndreaDistel, MartinPorat, Ze'evDivitini, GiorgioPostigo, Pablo AitorDjanashvili, KristinaPostnikov, PavelDolocan, AndreiPoulios, Ioannis G.Domb, AviPourrahimi, Amir MasoudDomenici, ValentinaPourret, OlivierDomine, MarceloPrades, Joan DanielDong, Cheng-DiPrado-Gotor, R.Doroudiani, SaeedPrashanth, Konda GokuldossDorozhkin, Sergey V.Prat, MariaDorval Courchesne, Noémie-ManuellePrati, SilviaDos Santos-García, A. J.Praus, PetrDosio, FrancoPredoi, DanielaDowell, Earl H.Prévot, VanessaDraheim, Roger R.Prida, Víctor ManuelDressel, MartinPrigiobbe, ValentinaDrory, Michael D.Prior, JoãoDu, FanfanProcek, MarcinDu, GuodongProcházka, MarekDu, ShangfengProchowicz, DanielDubey, AshishProfire, LenutaDumée, LudovicProslier, ThomasDumitrica, TraianPrudenzano, FrancescoDumont, Pierre J. J.Pryamikov, Andrey D.Duprez, DanielPuga, Alberto V.Durach, MaximPuglia, DeboraDutcher, DabrinaPulido, DavidDyatkin, BorisPulido, GerardoDziedzic, ArkadiuszPuppi, DarioDzimitrowicz, AnnaPuri, AnuDziubla, ThomasPutkonnen, MattiEbani, VelentinaPuttreddy, RakeshEbara, MitsuhiroPutz, Mihai V.Economou, DemetrePyatenko, AlexanderEda, ShigetoshiQi, DongchenEdgar, James H.Qian, Chunqi Edirisinghe, MohanQuémerais, BernadetteEgawa, YuyaQuijada-Garrido, IsabelEgelhaaf, Hans-JoachimQuintero, FélixEhrmann, AndreaQuirino, Joselito P.Einarsson, ErikRabczuk, TimonEklund, PerRabes, Blanca Del RosalEl Fray, MirosławaRadhakrishnan, SivaprakasamElaissari, AbdelhamidRaff, JohannesEl-Gendy, Ahmed A.Ragusa, AndreaElisabetta, EspositoRahier, HubertEl-Safty, Sherif A.Rai, AmriteshEmoto, MakotoRaikher, Yuriy L.Enculescu, MonicaRamanavicius, ArunasEndoh, TamakiRamírez-Rico, J.Ennas, GuidoRamos, DanielEnyashin, AndreyRamos, OscarErba, AlessandroRamos, PedroErdem, EmreRana, DipakEritja, RamonRao, AshitErné, Ben H.Raposo, MariaEscobedo, CarlosRaspanti, MarioEscorihuela, JorgeRastogi, Vibhore KumarEsposito, Emilio XavierRauch, ReinhardEsposito, GennaroRaudino, AntonioEstelle, PatriceRauwel, ErwanEstelrich, JoanRauwel, ProtimaEsteves da Silva, Joaquim C. G.Ravelli, DavideEtacheri, VinodkumarRazavi, PedramEugenio, María E.Razavi, S. M. J.Euler, William B.Read, JeffreyEuverink, Gert-JanReber, ChristianEvans, BenjaminRecek, NinaEvlyukhin, Andrey B.Reddy, AmaranathaEvtugyn, GennadyRegev, OrenEwels, Chris P.Reid, Alexander H. M.Fabiano, EduardoReinosa, Julián JiménezFabre, BrunoReipa, VytasFalco, AlbertoRemediakis, IoannisFalmbigl, MatthiasRescigno, AntonioFan, WeiRibot, Emeline J.Fang, JiyeRiccò, RaffaeleFaraldos, MarisolRichardson, JosephFare, SilviaRichardson, SimonFarrauto, Robert J.Rider, A. N.Fateixa, SaraRiela, SerenaFatouro, DimitrisRigano, DanielaFaustino, Maria A. F. Rigano, LuigiFavas, Paulo J. C.Righi, Maria CleliaFavre-Réguillon, AlainRim, You SeungFernandes, Mariana Rimondini, LiaFernandes, Susete NogueiraRinaldi, CarlosFernandez-Marchante, Carmen M.Ritmi, SamiFernández-Romero, Juan ManuelRivadeneyra, AlmudenaFernández-Sánchez, CésarRiveiro, Antonio Feron, KrishnaRizvi, SyedFerrante, CamillaRizzi, Gian AndreaFerrari, AldoRizzolio, FlavioFerrari, MicheleRoberts, NicholasFerraz, NataliaRodrigues, Alirio EgidioFerreira, Diana P.Rodríguez, AlejandroFerreira, Isabel M. M.Rodriguez, NoelFerrer, BelenRodríguez-Bernaldo De Quirós, AnaFerry, David K.Rodríguez-Castellón, EnriqueFiandra, LuisaRodríguez-Gómez, ArturoFidalgo De Cortalezzi, Maria M.Rodríguez-López, JoaquínFiedler, BodoRodriguez-Ropero, FranciscoFilgueira, CarlyRogl, Peter FranzFilip, PetrRojas, SergioFilon, Francesca LareseRojo, Maria AngelesFilpponen, IlariRomán-Martínez, M. C.Fiorito, SilvanaRomano, LuciaFischer, DagmarRomero, IsabelFischer, MichaelRoper, Donald KeithFischetti, Massimo V.Rosas, Ramiro RuizFleury, GuillaumeRosenholm, JessicaFlorczyk, Stephen J.Rossi, FilippoFlorea, IleanaRossignol, JeromeFlores-Arias, M. T.Rostovtsev, YuriFlox, CristinaRoth, Stephan V.Foldbjerg, RasmusRouhiainen, AriFologea, DanielRoussey, MatthieuFonseca, LuisRoy, AnupamFontana, Marc D.Roy, SagarForbes, ToriRoy, Utpal N.Formela, KrzysztofRozhkova, Elena A.Forment-Aliaga, AliciaRozynek, ZbigniewFornasiero, PaoloRubbens, AnnickFortunato, GiuseppinoRubini, SilviaFoster, Christopher W.Rubino, Federico MariaFrancesca, SelminRubio, Ramón G.Francia, CarlottaRuffino, FrancescoFrank, IrmgardRuffolo, Silvestro A.Frankcombe, TerryRuiz De Larramendi, IdoiaFranken, Nicolaas A. P.Ruiz, M. PilarFrasconi, MarcoRuiz-Rosas, Ramiro RafaelFratoddi, IlariaRumyantseva, Marina N.Frazzica, AndreaRupper, PatrickFresnais, JeromeRurali, RiccardoFrey, MargaretRussell, ThomasFriák, MartinRusso, Patrícia A.Fridman, MotiRusso, PietroFrigione, MariaenricaRusso, SaverioFrisenda, RiccardoRusu, CristinaFröhlich, EleonoreRyan, James G.Fromm, KatharinaRytter, ErlingFujii, TakenoriRyu, Du YeolFujita, Shin-ichiroSabalisck, Nanci PradoFukuda, TakeshiSabo, RonaldFulvio, Pasquale F.Sacchetti, AlessandroFuso, FrancescoSacco, AdrianoGaczynska, MariaSada, KazukiGadomski, AdamSadakane, MasahiroGaldiero, EmiliaSadki, SaïdGale, EricSadowski, ŁukaszGaleckas, AugustinasSadrzadeh, Mohtada Galetti, MariclaSafiuddin, Md.Galian, Raquel E.Sagawa, TakashiGalli, FedericoSahu, Saura C.Galstyan, VardanSaito, GenkiGambardella, AlessandroSaito, HidekazuGamble, Donald S.Saito, KazuyaGangishetty, MaheshSaitta, GaetanoGannot, IsraelSakaebe, HikariGarbovskiy, YuriySakai, JoeGarbuzenko, OlgaSakai, ToshioGarcía Calvo, José LuisSaladino, RaffaeleGarcía Ruiz, Francisco JavierSalado, ManuelGarcía, NuriaSalahub, DennisGarcía-Antón, JordiSalaoru, IuliaGarcia-Escorial, AsuncionSalaun, FabienGarcía-López, Elisa I.Salguero, Tina T.García-Martínez, Jesús-MaríaSalis, AndreaGarcía-Martinez, Joaquín C.Samaras, IoannisGardiner, PhilipSammelselg, VäinoGardner, Todd H.Samperi, FilippoGarno, JayneSancenón, FélixGarroni, SebastianoSánchez-García, DavidGartia, ManasSánchez-Royo, Juan FranciscoGaspar, VítorSandanayaka, Atula S. D.Gauden, Piotr A.Sandre, OlivierGebel, ThomasSangiovanni, DavideGeffroy, BernardSankarasubramanian, ShrihariGeise, GeoffreySanna, SimoneGeisler-Lee, JaneSano, TaizoGeissler, DanielSantamaría, AntonGeletii, YuriiSantini, AntonelloGendel, YuriSantos, AbelGenovese, DamianoSantos, João M.Gentile, PascalSaoud, KhaledGentile, PiergiorgioSapino, SimonaGeorge, Thomas F.Sarantopoulou, EvangeliaGeorgieva, RadostinaSarto, Maria SabrinaGeorgopanos, ProkopiosSashiwa, HitoshiGeso, MoshiSassoni, EnricoGhandi, KhashayarSastre-Santos, AngelaGhanotakis, Demetrios F.Sato, KatsuhikoGhidelli, MatteoSato, KimiyasuGhodake, GajananSato, TakayaGhorbani-Asl, MahdiSatriano, CristinaGhosh, AnindyaSavy, DavideGhosh, SohamSawada, HideakiGiacalone, FrancescoSawada, ToshikiGiannakas, ArisSberveglieri, VeronicaGiannakoudakis, DimitriosScala, AngelaGibson, ChristopherScalese, SilviaGiebeler, LarsScarabelli, LeonardoGigoux, VéroniqueScarano, DomenicaGil, Antonio Scarpa, MarinaGil-Mestres, AdriàScarselli, ManuelaGiménez-Marqués, MónicaScarso, AlessandroGindl-Altmutter, WolfgangScheicher, Ralph H.Gionco, ChiaraSchepdael, Ann VanGiordani, SilviaScheu, ChristinaGiorgi, LeonardoSchildgen, OliverGiosafatto, Concetta Valeria LuciaSchiraldi, DavidGiraud, Marie NoëlleSchirhagl, RomanaGirousi, StellaSchmedake, Thomas A.Giubileo, FilippoSchmid, MarkusGiuli, GabrieleSchmidt, Marek E.Giunchedi, PaoloSchneider, RaphaëlGiusti, IlariaSchöning, MichaelGizdavic-Nikolaidis, MarijaSchueneman, GregGnade, BruceSchwartzkopf, MatthiasGnade, Bruce E.Schwarz, KarlheinzGnyba, MarcinScott, Evan AlexanderGodinho, MariaScott, GeyerGoel, SauravScribante, AndreaGoerlitz, DetlefScrimin, PaoloGogneau, NoelleSebastian, VictorGoh, Kheng-LimSecu, MihailGoldoni, MatteoSeidel, JanGomez-Florit, ManuelSeifert, GotthardGómez-Graña, SergioSeisenbaeva , Gulaim A.Gómez-Ruiz, SantiagoSeker, ErkinGómez-Soberón, José ManuelSel, OzlemGómez-Tejedor, José AntonioSemaltianos, Nikolaos G.Gondi, Christopher S.Sengupta, BidishaGonzález Cortés, AraceliSenyuk, BohdanGonzalez, JoaquinSeo, Dae-ShikGonzalez, ZoraidaSeo, Young-SooGonzález-Sánchez, M. I.Seppälä, JukkaGoodarzi, MarjanSepúlveda, BorjaGordeeva, LarisaSepulveda-Escribano, AntonioGordiichuk, PavloSequeira, CésarGordillo, Jose ManuelSerpooshan, VahidGoreham, ReneeShacham-Diamand, YosiGorria, PedroShafirstein, GalGossuin, YvesShah, Nilam C.Govan, JosephShahjamali, Mohammad MehdiGovind Rao, VishalShahpari, MortezaGoycoolea, FranciscoShalev, GilGozdzicka-Jozefiak, AnnaShankar, EswarGrande Tovar, Carlos DavidShapiro, Bruce A.Granet, RobertShapter, JosephGranzow, TorstenSharifi, TivaGraule, ThomasSharma, AnjaliGraves, JosephSharma, JaswinderGray, DerekSharma, SwatiGreber, ThomasShehata, NaderGreco, Antonio Shemelya, CoreyGreenhalgh, DavidSher, PraveenGregor, IngoSheremet, MikhailGregory, JohnSherrell, PeterGrigsby, Christopher L.Shi, Jen-BinGrivel, Jean-ClaudeShi, WenboGrunwald, RüdigerShim, Min SukGryglewicz, GrażynaShimada, ToshihiroGuarrotxena Arlunduaga, Miren NekaneShimizu, KazuoGuénin, ErwannShimojo, MasayukiGueorguiev, G. K.Shimomura, TakeshiGueorguiev, Gueorgui K.Shin, HyunjungGuerrica-Echevarría, GonzaloShinya, NorioGuidotti, MatteoShrestha, Lok KumarGuillon, MarcSidhu, RohiniGuiot, CaterinaSiegel, JakubGunawidjaja, RayŠiffalovič, PeterGun'ko, YuriiSigg, Hans-ChristianGuo, QiuquanSignore, GiovanniGuo, ShuangzhuangSilva, Ana RosaGupta, MohitSilva, FilomenaGupta, Ram K.Silva, FilomenaGupta, SanjuSilva, HugoGupton, B. FrankSimmonds, Paul J.Guselnikova, Olga AndreevnaSimmons, TrevorGutleb, ArnoSimões, SóniaGuzman, MarceloSimon, SebastienHabuka, HitoshiSimonato, Jean PierreHadavinia, HomayounSimonsson, StinaHadjiargyrou, MichaelSimserides, ConstantinosHadjikakou, SotirisSing, JaiHadwiger, LeeSinha, JasmineHager, Martin D.Sinha, SudarsonHak, SjoerdSinha-Ray, SumanHakonen, AronSiniscalco, DarioHallberg, HåkanSiracusa, ValentinaHallett-Tapley, GenieceSitarz, MaciejHamad-Schifferli, KimberliSkirtach, AndreHamdana, GerrySłomkowski, StanislawHampton, JenniferSmått, Jan-HenrikHan, Dong-WookSmith, Henry I.Hanaor, DorianSmith, Robert E.Haniu, HisaoSoares, PaulaHanke, MichaelSobolev, KonstantinHano, ChristopheSobus, JanHanzu, IlieSoin, NavneetHaouas, MohamedSom, SudiptaHaponiuk, JózefSon, Seung UkHaraszthy, Violet I.Song, KenanHarding, IanSong, Young MinHariskos, DimitriosSonnier, RodolpheHaro, MartaSontakke, Atul DHarris, PeterSoria, SilviaHassan, Hassan M. A.Sotiropoulos, SotirisHassanin, HanySoucy, GervaisHatada, RurikoSoundappan, ThiagarajanHatakeyama, KazutoSpahn, ViolaHatziantoniou, SophiaSpasiano, DaniloHayase, MasanoriSpassov, SimoHein, JoachimSpeliotis, ThanassisHeise, BettinaSperber, Geoffrey H.Hejazi, VahidSpiess, LotharHelm, Christiane A.Spina, RobertoHémadi, MiryanaSpirk, StefanHempel, MartinSrivastava, G. P.Henry, MartinStadler, BethanieHeo, JinseokStamate, EugenHeo, KwangStan, Miruna SilviaHernandez, RebecaStano, PasqualeHerráez-Hernández, RosaStaruch, MargoHerrera-May, AgustínStassen, IvoHerrera-Melián, José AlbertoStavrakis, StavrosHerzig, PeterStavroulakis, Georgios E.Hess, Dennis W.Steenari, Britt-MarieHeuken, MichaelStefanou, GeorgeHeys, JeffreySteinfeldt, NorbertHigashi, NobuyukiStephan, DietmarHilleringmann, UlrichStephan, HolgerHinchet, RonanStępniowski, WojciechHinderliter, BrianStine, Keith J.Hippler, RainerStobiecka, MagdalenaHiramatsu, MineoStöckelhuber, Klaus WernerHirano, TomoyaStojek, ZbigniewHirsch, ThomasStolarczyk, AgnieszkaHirtz, MichaelStolojan, VladHjelme, Dag RoarStoychev, GeorgiHo, DanielStrankowski, MichałHo, Yi-ChengStrelniker, Yakov M.Hoath, StephenStreubel, RobertHoet, PeterStride, John ArronHohe, JoergStrozzi, MatteoHolade, YaoviStrunk, JenniferHolynska, MalgorzataSturm, E.Homaeigohar, ShahinStylianakis, MinasHong, SukjoonSu, ChunmingHorak, DanielSu, Hai-ChingHoriuchi, YuSuárez, IsaacHornak, JaroslavSuárez-Vázquez, Santiago IvánHorvath, DragosSubbiah, JegadesanHosier, Ian L.Suber, LorenzaHosmane, NarayanSubirats, XavierHotovy, IvanSubramani, ThiyaguHoung, BoenSuemune, IkuoHousecroft, CatherineSugiura, YukiHovorka, JanSuh, Won HyukHowarth, AshleeSujecki, SlawomirHowell, BobSukocheva, OlgaHoyos, PilarSumboja, AfriyantiHristoforou, EvangelosSumboja, AfriyantiHritcu, DoinaSun, HongyuHsieh, Ya-PingSun, Shih-JyeHsu, Yung-JungSun, WujinHu, MaocongSun, XiangchengHu, Shang-HsiuSürgers, ChristophHuang, Chih-ChiaSuzuki, ShinyaHuang, Chih-ChingSvetovoy, VitalyHuang, Chih-LingŚwietlik, RomanHuang, Genin GarySyrovy, TomasHuang, ianJangSysoev, VictorHuang, PeihuaSzczepanski, CarolineHuang, YingSzeghalmi, AdrianaHuczko, A.Szekely, GyorgyHueso, Jose L.Szewczyk, RomanHughes, Robert A.Szilágyi, IstvánHuminic, GabrielaSzulczewski, GregoryHung, I-MingTabib-Azar, MassoodHur, JaehyunTaboryski, RafaelHussain, TanveerTadanaga, KiyoharuHwang, Chi-SunTadmor, RafaelHwang, Suk-WonTaglietti, Angelo MariaHwang, YongsungTaheri, ShimaHytönen, VesaTaira, ShuIablokov, ViacheslavTakafuji, MakotoIannazzo, DanielaTakagi, NoriakiIatsunskyi, IgorTakagiwa, YoshikiIbáñez, MariaTakano, ToshiyukiIbrahim, ToniTakeoka, ShinjiIchikawa, TakahiroTakeshita, SatoruIchikawa, YoTakoudis, Christos G.Iconaru, SimonaTalaat, AhmedIda, JunichiTaler, JanIftekhar, SidraTamulevičienė, AstaIglesias, EmiliaTananaiko, OksanaIgnaszak, AnnaTangoulis, VassilisIjiro, KuniharuTański, TomaszInamura, KentaroTao, ShanwenInfantes Molina, AntoniaTaratula, OlenaIno, KosukeTasis, DimitriosInomata, KatsuhiroTavangarian, FariborzInsepov, Zinetula (Zeke)Tavares, AnaIoannides, TheophilosTavares, AnthonyIonete, Eusebiu IlarianTayeb, Ali H.Ionita, PetreTeghil, RobertoIsa, FabioTehranchi, AmirhosseinIsaacs, MarkTelegdi, JuditIsakov, DmitryTelukutla, Srinivasa ReddyIsasi Marín, Josefa Tempesta, MariaIshida, YoheiTeodorescu, FlorinaIshikawa, RyoTeodorescu, Valentin ŞerbanIshizaki, KenjiTeodoro, JoãoIslamoglu, TimurTercjaka, AgnieszkaIsoda, KatsuhiroTerry, IanIto, SeigoTesta, Maria LuisaIto, YoshokazuTeuvo, SuntioItoh, TamitakeTevetkov, NikolaiIvanda, MileTeymourian, HazhirIvanov, Ilia N.Thajudeen, ThaseemIvanov, IvanThakur, Vijay KumarIvanova, Elena P.Thang, Tran QuyetIzutsu, Ken-ichiThapliyal, VivekJacob, MohanThorat, NanasahebJadwicienczak, WojciechThünemann, AndreasJaeger, WolfgangThuo, MartinJagdale, PravinThuvander, MattiasJalowiecki-Duhamel, LouiseTibbetts, Katharine MooreJamróz, PiotrTiberto, PaolaJana, Sadhan C.Tiemann, MichaelJanas, DawidTilleu, RichardJanát-Amsbury, Margit M.Tiseanu, CarmenJang, ChangheuiTiwari, DevendraJang, Ho WonTiwari, Rakesh K.Jang, Jae EunTiwari, SanjayJankauskaite, VirginijaToda, MasayaJariwala, DeepTokunaga, EijiJarvis, KarynTolkou, AthanasiaJasieniak, JacekTomasini, ClaudiaJavier, Sánchez-NievesTomer, Vijay K.Jayant, Rahul DevTomita, ShunsukeJędrzejczyk, Roman J.Toncelli, ClaudioJelliss, Paul A.Tongay, SefaattinJeong, Chang KyuToplijska, AntoniaJeong, Mun SeokTopoglidis, EmmanuelJeong, Nak Cheon Torigoe, KanjiroJesionowski, TeofilTorimoto, TsukasaJeyadevan, BalachandranToro, Roberta G.Jiang, KeToroker, Maytal CasparyJiang, XuchuanTorrado, EgídioJicsinszky, LászlóTorre, Maria LuisaJimenez Ballesta, Ana EvaTorrens, FranciscoJiménez-Aparicio, ReyesTorres Lagares, DanielJimenez-Villacorta, FelixTournie´, EricJing, QingshenTowner, Rheal A.Jiu, JintingTownley, Helen E.Jmerik, V. N.Toyao, TakashiJohn, ŁukaszTran, CuongJohnson, DavidTrinh, ThuatJohnson, ShaneTripathi, Jitendra KumarJordan, AlexTripathi, KumudJoshi, TanmayaTrivedi, RahulJou, ShyankayTrivellin, NicolaJulien, Christian M.Trukhanov, Alex V.Jun, Bong-HyunTsamis, ChristosJung De Andrade, MônicaTseng, I-HsiangJung, Ho-SupTseng, Wei-LungJung, In HwaTsibidis, GeorgeJung, SeunhoTsigaridas, GeorgiosJuodkazis, SauliusTsilipakos, OdysseasJurado, Amalia S.Tsitsilianis, ConstantinosJurado-Sánchez, BeatrizTsitsilonis, Ourania E.Jursich, GregoryTsoukalas, DimitriosJuskenas, RemigijusTsubota, ToshikiKaczmarek, Anna M.Tsuji, YasuhideKahlert, HeikeTsurkan, Mikhail V.Kakkar, AshokTsutsui, MakusuKako, TetsuyaTsutsumi, OsamuKalali, Ehsan Naderi Tsuyumoto, IsaoKalantar-Zadeh, KouroshTulliani, Jean-MarcKalbáčová, Marie HubálekTung, Tran ThanhKalhapure, RahulTzounis, LazarosKalinich, JohnUccelletti, DanielaKallitsis, Joannis K.Uchihashi, TakashiKalogiannis, Konstantinos G.Ueda, TadaharuKamali, SaeedUeno, TakafumiKamaly, NazilaUezu, KazuyaKamimura, TakafumiUgo, PaoloKamińska, AgnieszkaUjihara, MasakiKamiński, MarcinUji-i, HiroshiKamp, MarlousUllah, AmanKampouris, DimitriosUm, HandonKanda, KazuhiroUmehara, NoritsuguKang, ChulhunUmeton, CesareKang, HyoUmrath, StefanKang, Jun-GillUrata, ChihiroKang, YoungsooUrbano, Ana MargaridaKanik, MehmetUsui, HidetomoKano, ShinyaVahabi, HenriKantartzis, Nikolaos V.Vaiano, VincenzoKappagantula, KeertiVaidya, BhuvaneshwarKarabchevsky, AlinaValle-Delgado, Juan JoséKaragiannakis, GeorgeVallee, ChristopheKarahan, Hatice AylinVan Pomeren, MarindaKarapanagiotis, IoannisVanEpps, J. ScottKarasawa, SatoruVankayala, RavirajKarczewski, GrzegorzVaquero, JavierKargarzadeh, HaniehVargas, ZulemaKarnahl, MichaelVarma, Rajender S.Karnati, PriyankaVasilakaki, MariannaKartopu, GirayVasile, Bogdan StefanKartsonakis, IoannisVasile, CorneliaKatunin, AndrzejVasquez, YolandaKaushik, AjeetVasta, SalvatoreKawao, NaoyukiVautard, FredericKawasaki, HideyaVaz, CarlosKazakov, SergeyVázquez De Aldana, Javier RodríguezKazek-Kęsik, AlicjaVega, VictorKeegan, Kirk A.Venditti, IoleKelarakis, AntoniosVenkatraman, VishweshKelly, RobertVerbeek, Jos H.Kennedy, JohnVerdejo, BegoñaKerch, GarryVerdolotti, LetiziaKessel, DavidVereshchaka, Alexey A.Kessler, VadimVerma, SannyKevadiya, BhaveahVertruyen, BenedicteKhadka, Dhruba B.Vetter, Stefan W.Khan, Mohammad A.Vicenzo, AntonelloKhandaker, MorshedVideira, Romeu A.Khisamutdinov, EmilVidor, Fábio F.Khodadadi, Jay M.Vieru, DumitruKhodadoust, Amid P.Vila, AnnaKidoaki, SatoruVilanova, XavierKiisk, ValterVilaseca, FabiolaKikuchi, HiroakiVilela, Diana Kim, DaekeunVilla, AlbertoKim, DaewonVillares, AnaKim, Dai-SikVillegas, María-ÁngelesKim, Do YoungViñas, MiguelKim, DonginVismara, ElenaKim, Hak-YongViter, RomanKim, Hee-SeokVitiello, RosaKim, HojoongVittorio, OrazioKim, JaeyunViva, Federico A.Kim, JaheonVivo, PaolaKim, JeonghunVizireanu, SorinKim, Jong HyunVlachou, MarilenaKim, Jung KyuVlǎdoiu, RodicaKim, Jun-HyunVo, MinhKim, Keun SuVohra, VarunKim, Kyong HonVohra, Yogesh K.Kim, Moon IlVoicu, Ştefan IoanKim, Myung JunVoiry, DamienKim, Myun-SikVoliani, ValerioKim, Seong YunVolk, DavidKim, Soo YoungVolkov, Valentyn S.Kim, SunkookVolkov, YuriKim, SunwookVotruba, MarcelaKim, YounghunVozzi, GiovanniKim, Young-KwanVrana, Nihal EnginKim, YoungmeeVriesekoop, FrankKiss, JánosVul, AlexanderKitahama, YasutakaWagner, ThorstenKitamura, TakayukiWal, R. L. VanderKityk, IwanWalsh, FrankKiwi, Jolin T.Walsh, JamesKlajnert-Maculewicz, BarbaraWang, BinghaoKlinkova, AnnaWang, GaoxueKliros, George SWang, ShengnianKlotz, MichaelaWang, TaoKnopp, DietmarWang, XiKo, Dong KyunWang, XianqiaoKo, Jang MyounWang, XupKo, SeungWang, YingKobayashi, NoriyukiWang, YueKobayashi, ShunsukeWang, Yun-CheKoetz, JoachimWang, ZhantongKoinkar, PankajWatanabe, MasatoshiKojima, ToshikatsuWatanabe, MotonoriKokado, KentaWatras, AdamKoksharova, OlgaWee, Lik H.Kolasinski, Kurt W.Wei, Da-HuaKolpashchikov, DmitryWei, GangKonarova, MuxinaWei, ZhishunKonegger, ThomasWeiss, Clemens K.Konstantinov, Spiro M.Wen, Amy M.Konstantynovski, KostyantinWeng, QunhongKoohbor, BehradWertz, Philip W.Kopel, PavelWhite, Jason C.Kordas, KrisztianWhite, MarkKordulis, ChristosWiliams, VanceKörstgens, VolkerWillerth, Stephanie M.Kortaberria, GalderWilliams, GarethKoshimizu, MasanoriWilson, Andrew J.Kost, GeraldWimbush, StuartKostoglou, MargaritisWinkler, David A.Kosuke, SugawaWiśniewski, MarekKotobuki, MasashiWitek, LukaszKou, TianyiWitkowski, ArturKoukouzas, NikolaosWitte, HartmutKoumoulos, Elias P.Wittmann, FolkerKoutselas, IoannisWojcieszak, RobertKoutsogeorgis, DemosthenesWojnar, PiotrKovalcik, AdrianaWojnarowicz, JacekKovalenko, SergeyWolverson, DanielKovalevsky, Andrei V.Wong, Danny K. Y.Kowalska, EwaWong, EugeneKozyukhin, Sergey A.Wu, R. J.Krawiec, MariuszWu, Sheng YunKriegel, IlkaWu, TaoKriston, AkosWujcik, EvanKröger, RolandWuttke, StefanKrolczyk, GrzegorzWycisk, RyszardKroll, PeterXi, WeixianKruger, Jacob S.Xia, TianKryschi, CarolaXia, WenjieKu, Bon-CheolXie, KunpengKubiak, TomaszXu, QiangKubo, MasatakaYadavali, SagarKubo, TakanoriYakimov, EugeneKubota, KeiichiYamada, ToruKubota, YasuhiroYamaguchi, SeijiKudelski, AndrzejYamamoto, AkiraKudryashov, Sergey I.Yamamoto, GoKujala, SamiYamamoto, YusukeKujawa, JoannaYamazoe, NoboruKujawski, WojciechYan, XuefengKukli, KaupoYanagida, SayakaKulinich, SergeiYang, GuangKumada, TakayukiYang, HangshengKumar, AdityaYang, Rong-SenKumar, ManishYang, Ta-IKumar, PawanYang, Teng-ChunKumar, PrasunYarowsky, PaulKumar, RajYasui, KyuichiKumar, ShaileshYates, Matthew Z.Kumar, VivekYe, YanqiKumeria, TusharYeh, Min-LongKumi-Diaka, JamesYehezkeli, OmerKundu, JoydipYen, Hung-JuKundu, ManabYentekakis, Ioannis V.Kuo, Shiao-WeiYerushalmi-Rosen, RachelKurlyandskaya, Galina V.Yi, JiabaoKuroda, YoshiyukiYim, ChangyongKusano, YukihiroYoon, Dae-HoKusiak-Nejman, EwelinaYoon, HyeonseokKustov, L. M.Yoon, HyunKuttner, ChristianYoon, In-SooKuzhir, PavelYoon, MinjoongKuzuya, AkinoriYoon, OkjaKvítek, LiborYoshimoto, MasahiroKwon, Hyuck-InYou, JungmokKwon, Soon-HongYounesi, RezaKyzas, GeorgeYu, HakkiLa Deda, MassimoYu, Hsin HerLa Parola, ValeriaYu, Ki JunLacarbonara, WalterYu, MeihuaLächelt, UlrichYu, TaekyungLachman, NoaYu, Yuan-HsiangLacroix, DavidYuba, EijiLadavos, Athanasios K.Yusenko, Kirill V.Lagrow, Alec P.Zabihi, OmidLähnemann, JonasZablotskii, VitaliiLai, Jui-YangZaccheroni, NelsiLai, StefanoZaitsev, AlexanderLai, Yi ShengZambon, DanielLaity, Peter R.Zangari, GiovanniLambrecht, Daniel S.Zarrelli, MauroLamprou, DimitriosZdarta, JakubLan, YuchengZeinert, AndreasLancaster, Simon J.Zeiri, LeilaLandi, GianlucaZeng, HuangLange, HolgerZhang, JingjingLanger, Jerzy J.Zhang, LaichangLao, Ka UnZhang, LibingLari, LeonardoZhang, RunLaromaine, AnnaZhang, XianghuiLarraneta, EnekoZhang, Xueqiang AlexLartigue, LénaïcZhao, BoteLatorrata, SaverioZhao, HangboLatterini, LoredanaZhao, XinxinLattuada, MarcoZhao, YudaLaukkanen, PekkaZheng, KaiboLaurenzana, AnnaZheng, XintingLavina, SandraZhilyaev, AlexanderLazanu, SorinaZhong, Yu LinLazzara, GiuseppeZhou, DezhongLe, DuyZhou, ShengqiangLe, HuirongZhou, YouLebeau, LucZhu, WeiLebedev, Oleg IgorevichZhu, YanqiuLeBlanc, GabrielZi, YunlongLeblond, JeanneZielińska-Jurek, AnnaLedo, AnaZille, AndreaLee, BongmookZimmerman, William B.Lee, Byung YangZiółek, MarcinLee, Chia-HungZoltán, PásztiLee, Chul-HoZou, QiongjingLee, Dong HyunZsuzsanna, SzallerLee, Hee-JoZuluaga, Hector Fabio Lee, IlkeunZumbrunnen, David A.Lee, Jaehwang


